# Posterior shoulder dislocation with associated reverse Hill-Sachs lesion: treatment options and functional outcome after a 5-year follow up

**DOI:** 10.1186/s12891-017-1808-6

**Published:** 2017-11-13

**Authors:** Markus Guehring, Simon Lambert, Ulrich Stoeckle, Patrick Ziegler

**Affiliations:** 1 Department for Traumatology and Reconstructive Surgery, BG Trauma Center Tübingen, University of Tübingen, Schnarrenbergstr 95, 72076 Tuebingen, Germany; 2 Shoulder and Elbow Service, Royal National Orthopaedic Hospital, Stanmore, HA7 4LP UK

**Keywords:** Posterior shoulder dislocation, Defect size, Osteosynthesis, Outcome

## Abstract

**Background:**

The current study describes several surgical techniques for the treatment of the reverse Hill - Sachs lesion after posterior shoulder dislocation; we also aimed to present long term results followed for a minimum of five years.

**Methods:**

This study is a prospective case series of 17 patients who were treated in our clinic between 2008 and 2011. Patients with a defect size smaller than 25% of the articular surface were treated conservatively. An endoprosthesis of the glenohumeral joint was implanted in patients with a defect size bigger than 40%. All remaining patients were treated by a variety of operative techniques, depending on the quality of the bone and size of the defect.

**Results:**

Twelve of seventeen patients had a defect size of the humeral articular surface between 25% and 40% with a mean age of 39 years. Depending on the defect size these patients were treated with retrograde chondral elevation, antegrade cylindrical graft or a graft of the iliac bone crest with an open approach. All the procedures showed fair results, e.g. the open approach with a graft of the iliac bone crest (2010: Dash 3.89, Constant 90.33, Rowe 86.67; 2015: Dash 2.22, Constant 92.00, Rowe 93.33).

**Conclusion:**

The open approach is not a disadvantage for the functional outcome. The treatment algorithm should involve the superficial size of the defect as well as the depth of the defect and the time interval between the dislocation and the surgical treatment.

**Trial registration:**

223/2012BO2, 02 August 2010.

## Background

Posterior shoulder dislocation is a rare injury, comprising 2% to 5% of all shoulder dislocations [1, 2] and up to 10% in patients with shoulder instability (mostly polar type II and III according to the Stanmore instability classification). The spectrum of posterior dislocation ranges from acute traumatic dislocation to chronic irreducible dislocations, and in combination with a proximal humeral fracture [3]. An extreme muscle contraction (seizures or electric shock), a direct or indirect trauma that occurs with flexion, adduction and internal rotation of the affected arm, is pathognomonic for the posterior shoulder dislocation [4–6].

Cooper first described the typical clinical signs of the posterior shoulder dislocation: dorsal protrusion of the humeral head in accordance with a flattened front shoulder and prominent coracoid, significantly limited or even repealed external rotation, or fixed internal rotation and restricted abduction under 90 degrees [7]. However, in contrast to the anterior shoulder dislocation, there may be very little obvious deformity of the shoulder girdle. Accordingly, the posterior shoulder dislocation is not detected in the primary examination in 60% to 79% of the cases [1, 2, 8]. Periods of over 10 years between dislocation and diagnosis are described in the literature [4].

**Fig. 1:**
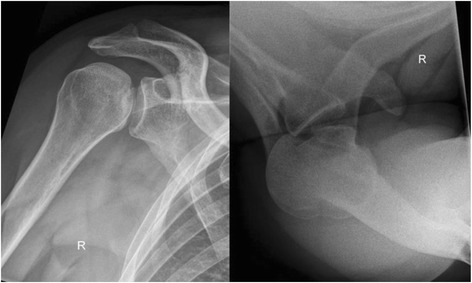
Axial and ap view of posterior shoulder dislocation

**Fig. 2:**
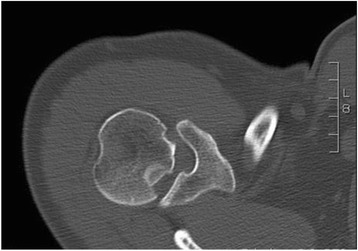
CT scan after posterior shoulder dislocation

A radiological examination in two views is obligatory (anteroposterior (a-p) and axial; Fig. 1). If pain precludes an axial x-ray because of limited abduction, a ‘scapular-Y’ view is recommended, even if there is marked pain. In the a.-p. view the posterior dislocation classically appears as a ‘light-bulb’ but this is not diagnostic and dislocation is thus sometimes difficult to detect [9]. Moreover, a careful clinical examination (lack of external rotation in a patient with a history of a shoulder injury) is mandatory. Computed tomography (CT) is essential for evaluating the injury and for preoperative planning regarding bone defects in the humeral head (Fig. 2). A magnetic resonance imaging scan (MRI), with contrast, is useful to diagnose lesions of the labrum and rotator cuff [1, 2, 4], particularly of the incarcerated tendon of the long head of biceps in irreducible dislocations [4]. Compared to anterior shoulder dislocations with defects in the anterior labrum and capsule (that is, soft tissue lesions), posterior dislocation typically causes bone lesions (the anterior humeral head impression fracture,, otherwise known as the “reverse Hill-Sachs lesion”, McLaughlin lesion, or “l’encoche de Malgaigne”) [5]. Other injuries such as lesions of the posterior labrum, or fractures the posterior glenoid rim are described [10–12]. Treatment depends on the size of the bone defect, the duration of the dislocated condition, and the functional demand of the patient [13].

Conservative treatment is possible with a stable situation after closed reduction and no significant bone defect. Subsequently, the affected shoulder should be immobilized in internal rotation or neutral position over a short period of time [6, 14]. Depending on the size, the reverse Hill-Sachs lesion is a risk factor for re-dislocation and therefore a surgical treatment is normally recommended [15]. For the treatment of the bone defect in the region of the humeral head, a variety of surgical procedures are described in the literature: filling the defect by tendon transposition of the subscapularis muscle [5], medial transposition of the lesser tuberosity [16] or allograft [17]; rotational osteotomy [18]; and hemi- or total arthroplasty [19, 20] are options.

The choice of the surgical technique depends on the size of the bone defect. With a stable shoulder joint and a defect of less than 25% of the articular surface, conservative treatment normally shows a satisfactory outcome. Reconstruction of the anatomical joint surface is recommended for defects between 25% and 40% of the articular surface. Lesions with greater defects than 40% of the articular surface should be treated with shoulder prosthesis [16, 19]. The literature of posterior dislocations of the shoulder largely comprises case reports or small series, while studies with a significant number of patients are rare. The aims of the present study are: to evaluate the anatomical reconstruction of the articular surface in a homogeneous patient population; and to evaluate the long-term functional outcomes in the cohort.

## Methods

Between January 2008 and December 2011, 17 patients were treated with a posterior shoulder dislocation. The diagnosis was confirmed by using two orthogonal x-rays of the shoulder joint (anteroposterior (AP) and axial views). Closed reduction of the dislocation was attempted immediately under analgesia and sedation. A CT scan, to evaluate the size of the reverse Hill-Sachs lesion, was undertaken if closed reduction was not possible using the method of Cicak et al [21]. Five patients were excluded from this study. Four patients with defects of less than 25% of the articular surface in whom the joint was stable after open reduction were treated conservatively. One patient with a defect greater than 40% of the articular surface had a total shoulder arthroplasty. The remaining twelve patients had a reverse Hill-Sachs compression fracture involving 25–40% of the articular surface of the humeral head following a traumatic posterior shoulder dislocation.

All patients were male with a mean age of 39 years (range 17–55). The postoperative results were evaluated after a mean of one and five years following intervention using the Constant score [22], the Rowe score [23] and the DASH (disability of the arm, shoulder and hand) score [24]. The subjective perception of pain was evaluated by a VAS (visual analogue scale). No patient had multidirectional instability, prior shoulder surgery, or a neuromuscular disorder.

**Fig. 3:**
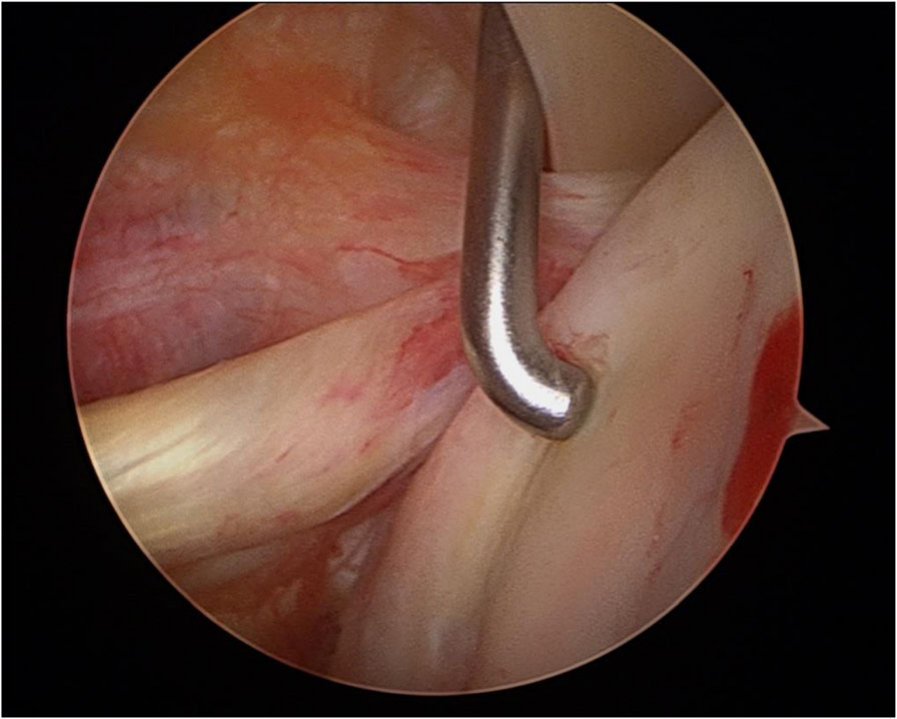
Diagnostic arthroscopy after posterior shoulder dislocation to detect cartilage defects

**Fig. 4:**
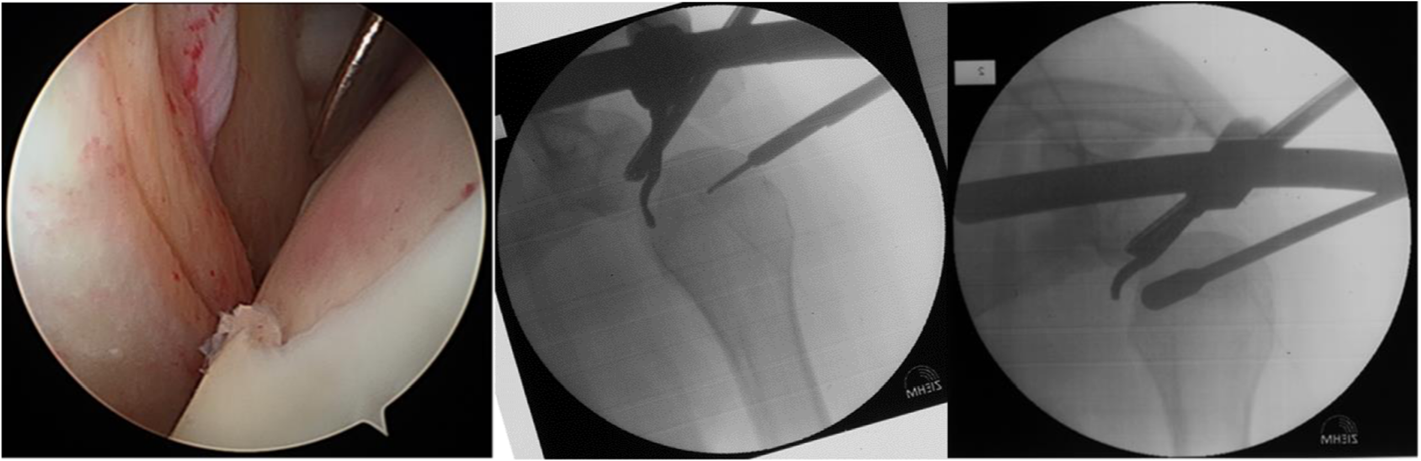
Arthroscopic retrograde elevation with target device from cruciate ligament surgery

**Fig. 5:**
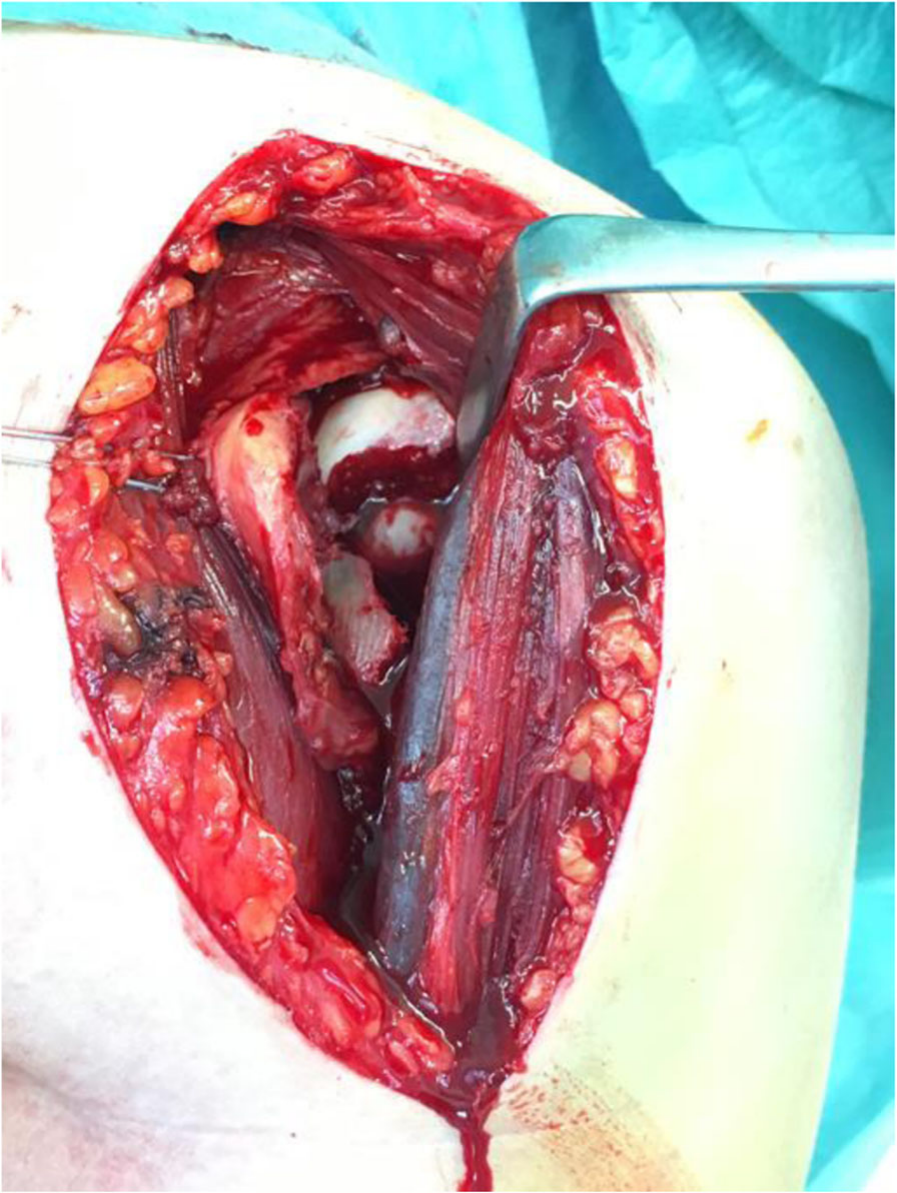
Open approach for the treatment with an iliac bone crest graft

**Fig. 6:**
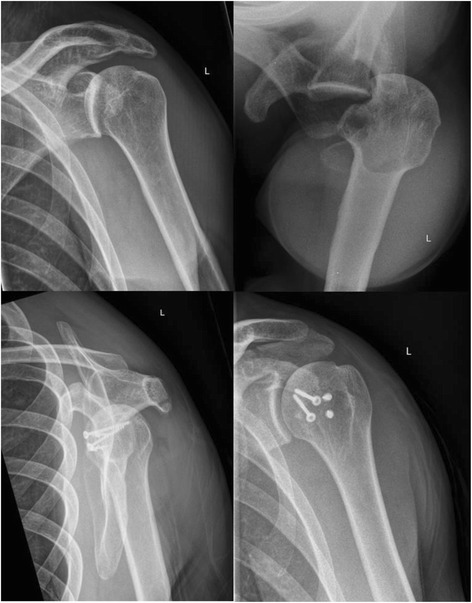
Before and after reconstruction with an autologous graft of the iliac crest with small fragment screws

**Fig. 7:**
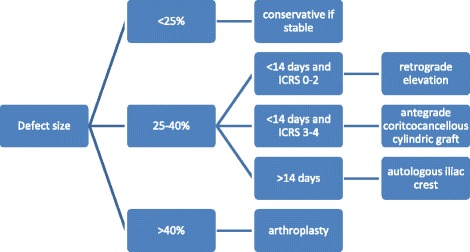
Treatment algorithm for posterior shoulder dislocations depending on defect size and timer interval between the trauma and surgery

Diagnostic arthroscopy of the affected shoulder was attempted in all cases. The depth of the bone defect and the cartilage of the humeral articular surface were noted, together with associated injuries of the labrum and the rotator cuff. If no deep lesions of the cartilage surface were detected during the diagnostic arthroscopy (ICRS classification grade 0–2, Fig. 3) and the time between the shoulder dislocation and the operative treatment was less than 14 days, we elected to restore the joint surface by retrograde elevation with arthroscopic assistance using a target device from the knee ligament surgery (Fig. 4). Larger cartilage lesions (ICRS classification grade 3 + 4) were treated during open debridement [25] using a delto-pectoral approach in all cases (Fig. 5). If the interval between the accident and operative treatment was less than 14 days the joint surface was reconstructed with antegrade cortico-cancellous cylindrical grafts. If the interval was more than 14 days the defect was reconstructed using an autologous iliac crest fixed by small fragment screws (Fig. 6). The therapeutic algorithm is shown in Fig. 7.

### Statistical analysis

Statistical analysis was performed in SPSS (version 22.0, SPSS Inc., Chicago, US). The t-test was used to calculate differences between the one and five year evaluations of pain and function. Differences in outcome between the surgical techniques were calculated using the Kruskal-Wallis variance method.

## Results

The cause of the posterior shoulder dislocation was a high energy trauma in 75% (8 cases). The average length of in-hospital stay was 7.6 (4-24) days (Table 1).

**Table 1:** Epidemiological data of the patients included in the study

Criteria	Specification	Total	
Total number of patients	Reverse Hill-Sachs lesion[n]	12	
Age	total [y (range)]	39 (17–55)	
Gender	male [n]	12	100%
	female [n]	0	0%
Treatment	Arthroscopic reduction and retrograde elevation [n]	5	42%
	Open reduction antegrade cylindric graft [n]	4	33%
	Iliac bone crest	3	25%
Cause of injury	High energy trauma [n]	9	75%
	Low energy trauma [n]	2	17%
	Eplilepsy [n]	1	8%

In five cases with an ICRS score of 0–2 (42%), arthroscopically-assisted elevation of the articular surface was performed. Four patients had ICRS grade 3 or higher cartilage lesions, and were treated by antegrade cortico-cancellous cylindrical grafts. Three patients had polytrauma and had definitive treatment of the shoulder more than 14 days after injury using iliac crest cortico-cancellous graft. There were no postoperative infections, bleeding or nerve injuries, and no complication after harvesting the iliac bone graft. There were no re-dislocations over the period of review.

A complete minimum follow up of five years was achieved in all patients. There was an improvement in outcomes at five years compared with the one year results: 5.28 points in the DASH score, 7.58 points in the Constant score, 8 points in the ROWE score and 0.86 points on the VAS on average (Table 2). Patients who were treated with a corticocancellous graft of the iliac crest had the best results after one and five years within the small group of patients (year one: Dash 3.89, Constant 90.33, Rowe 86.67; year five: Dash 2.22, Constant 92.00, Rowe 93.33). This could be demonstrated in all evaluated scores as well as in the VAS (year one, 0.67; year five, 0.5) (Tables 3 and 4).

**Table 2:** Functional outcome and the VAS after one and five years showed better results in the five year follow up in all evaluated scores

Score	2010	2015
DASH	10.49 ± 2.57	5.21 ± 1.37
Constant	81.92 ± 3.10	89.50 ± 2.72
ROWE	72.92 ± 5.56	87.92 ± 3.61
VAS	1.67 ± 0.36	0.81 ± 0.19

**Table 3:** Functional outcome and the VAS showed the best results for patients treated with an iliac bone crest graft in 2015

Score 2015	Retrograde elevation (*n* = 5)	Antegrade cylindric graft (*n* = 4)	Iliac bone crest (*n* = 3)	p
DASH	7.33 ± 2.64	4.79 ± 2.02	2.22 ± 1.21	.39
Constant	89.80 ± 4.66	87.25 ± 5.59	92.00 ± 4.61	.84
ROWE	85.00 ± 7.25	87.50 ± 6.29	93.33 ± 3.33	.89
VAS	0.94 ± 0.30	0.88 ± 0.43	0.50 ± 0.29	.67

**Table 4:** Functional outcome and the VAS showed the best results for patients treated with an iliac bone crest graft in 2010

Score 2010	Retrograde elevation (*n* = 5)	Antegrade cylindric graft (*n* = 4)	Iliac bone crest (*n* = 3)	p
DASH	12.17 ± 4.21	13.33 ± 5.28	3.89 ± 0.56	.18
Constant	79.00 ± 3.70	79.25 ± 7.33	90.33 ± 2.33	.31
ROWE	76.00 ± 8.57	58.75 ± 8.00	86.67 ± 8.33	.11
VAS	1.80 ± 0.37	2.25 ± 0.85	0.67 ± 0.33	.21

## Discussion

Optimal treatment of the reverse Hill-Sachs lesion after posterior shoulder dislocation remains controversial. Due to the rare entity of this injury pattern, high numbers of cases in clinical trials are difficult to generate [17, 26]. The cause of traumatic, non-epileptic posterior dislocation is usually a direct force applied to the adducted and extended arm in internal rotation [3] while the mechanism in epilepsy is considered to be a high muscular force generating internal rotation in an adducted arm [27, 28]. Posterior dislocation of the humeral head may cause a posterior-directed shearing of the labrum or the bony glenoid rim [29, 30] but is primarily characterized by the osteochondral impression fracture of the ventromedial articular surface of the humeral head, the so-called reverse Hill-Sachs lesion [5, 31]. Concomitant neurovascular injuries or lesions of the rotator cuff occur much rarely after posterior dislocation [30, 32]. We observed two lesions of the labrum requiring operative treatment in addition to the reverse Hill-Sachs lesion in this study. These were detected during the diagnostic arthroscopy and accordingly fixed by suture anchors. We did not detect any rotator cuff injuries.

The treatment of posterior instability with a reverse Hill-Sachs lesion considers the arc of stability relative to the arc of rotation of the humeral head with respect to the glenoid surface. The treatment therefore largely depends on the size of the humeral head defect [33]. The surgical strategies are either: the optimization of the surface arc of rotation by restoration of the sphericity of the humeral head (and thereby optimizing the arc of stability), or the restriction of motion of the humeral head relative to the glenoid so that the arc of stability becomes equivalent to the more limited arc of rotation. Various techniques have been described to reconstruct the joint surface defect by osteochondral allograft [34]. Miniaci and Gish performed osteochondral transplantation using fresh-frozen, size-matched allograft in 18 patients with a defect greater than 25% with an average follow-up of 50 months. The allografts were fixed with Kirschner wires [35]. Outcomes were reasonable with an average Constant score of 78.5 points. Several complications such as osteoarthritis, secondary sintering, subluxation and wire migration were noted. In another series, Diklic et al. recorded an average Constant score of 86.8 points with a follow-up period of 54 months after reconstruction using femoral allograft and fixation with cannulated screws [36]. Gerber and Lambert showed an average Constant score of 82 points in a group of 4 patients after reconstruction of the articular surface by femoral allograft [17]. Krackhart et al. recommended fixing the subscapularis tendon with suture anchors into the defect [37]. This leads to restriction of internal rotation [38].

Only patients with a defect size between 25 and 40% of the joint surface after posterior shoulder dislocation were included in our study. The patients were reviewed at a mean of one year and five years after surgery. Irrespective of the operative technique used in the present study, we observed a fair outcome with a mean Constant score of 81.92 and 89.50 points respectively. Over time, all of the scores showed an improvement, with low pain scores, related to exercise, at both time points. The best outcome for patients at both time-points was observed after using an autologous iliac crest cortico-cancellous bone graft. This could result from a lower secondary sintering rate of cortico-cancellous bone graft compared to retrograde elevation of the articular surface or antegrade cylindrical osteochondral grafting. These findings have to be interpreted carefully due to the small number of cases of this study.

The limiting factor of this study remains the small number of cases. Nevertheless, we believe the treatment algorithm shown in Fig. 7 is very useful, since it includes the extent of cartilage damage and the interval between the injury and surgical treatment, in addition to the size of the humeral defect.

## Conclusion

This study shows the results and techniques of reconstructive treatment options for reverse Hill-Sachs lesion after posterior shoulder dislocation. The best results were demonstrated in the reconstruction of the joint surface by autologous iliac crest grafts. The open approach does not appear to be a disadvantage for the functional outcome despite the invasiveness. In our opinion, the treatment algorithm of the reverse Hill - Sachs lesion should involve the superficial size of the defect, as well as the depth of the defect and the time interval between the dislocation and the surgical treatment.

## Abbreviations

CT: Computed tomography
DASH: Disability of the arm, shoulder and hand
ICRS: International Cartilage Repair Society
MRI: Magnetic resonance imaging
SD: Standard Deviation
VAS: Visual analogue scale
